# Laparoscopic surgery of a presacral epidermoid cyst: A case report

**DOI:** 10.1016/j.ijscr.2019.04.043

**Published:** 2019-05-04

**Authors:** Koichi Mohri, Tadahiro Kamiya, Kazuhiro Hiramatsu, Yoshihisa Shibata, Motoi Yoshihara, Taro Aoba, Akira Ito, Takehito Kato

**Affiliations:** Department of General Surgery, Toyohashi Municipal Hospital, Toyohashi, Aichi, Japan

**Keywords:** CT, computed tomography, MRI, magnetic resonance imaging, Presacral tumor, Developmental cyst, Epidermoid cyst, Case report

## Abstract

**Introduction:**

The best approach for resecting epidermoid cysts is still controversial. We describe a case of an epidermoid cyst in which laparoscopic resection was performed successfully.

**Presentation of case:**

63 × 55-mm well-defined cystic mass was incidentally detected by computed tomography in the presacral cavity of a 50-year-old woman during evaluation for upper abdominal pain. Magnetic resonance imaging showed a cystic tumor with a low signal intensity on T1-weighted images and, high signal on T2-weighted images in the left dorsal side of the rectum. This tumor was diagnosed as a developmental cyst, and laparoscopic resection was performed. Resection of the tumor was performed with negative margins. This tumor was histopathologically diagnosed as an epidermoid cyst. There was no evidence of malignancy, and no postoperative event or signs of recurrence occurred 6 months postoperatively.

**Discussion:**

In our patient, there was no difficulty in the field of view and forceps operability during laparoscopic surgery. Furthermore, it is possible to perform laparoscopic surgery with minimal damage to the muscles, nerves, and rectum, leading to the preservation of anal function.

**Conclusion:**

Laparoscopic resection of an epidermoid cyst may be a better option in carefully selected cases with consideration of the tumor size and location.

## Highlights

• The best approach for resecting epidermoid cysts is still controversial.
• We successfully performed laparoscopic resection of an epidermoid cyst.
• Laparoscopic resection may be a better option in carefully selected cases.
• However, the tumor size and location must also be considered.

## 1 Introduction

Epidermoid cysts that developing the presacral cavity are rare congenital cystic tumors. They are mostly benign tumors, but they reportedly have risks of infection, fistula, and malignancy [1]. Therefore, surgical resection is recommended. Their anatomical location makes them difficult to reach, so they have traditionally been accessed through a posterior or transabdominal approach. The best approach is still controversial. Recently, a successful laparoscopic approach has been reported [2]. Laparoscopic surgery can provide magnification of the visual field and reachability into the deep pelvis with forceps. It is possible to perform surgery with laparoscopy more safely and less invasively. Herein, we describe a case of an epidermoid cyst in which laparoscopic resection was performed successfully. This case report has been written in line with the SCARE criteria [3].

## 2 Presentation of case

**Fig. 1:**
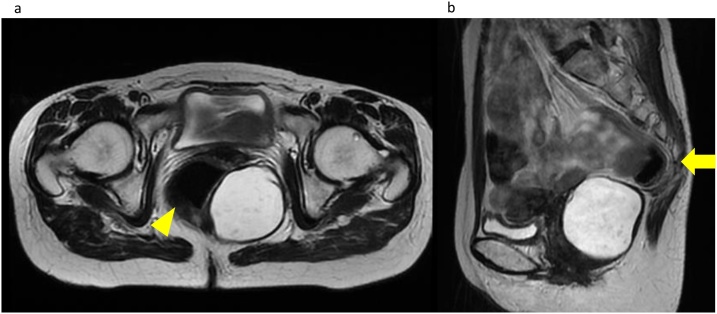
Magnetic resonance imaging shows a unilocular cystic tumor with a high signal intensity on T2-weighted images in the pelvis. (a) A well-defined homogeneous cystic mass is located in the left dorsal side of the rectum (arrow head) and (b) ventral side of the coccyx (arrow).

A 50-year-old woman was referred to our hospital for further examination of a cystic tumor in the pelvis that was incidentally detected by computed tomography (CT) during evaluation for upper abdominal pain. She had no past medical and surgical history. On digital rectal examination, a soft mass was palpated outside of the rectal wall. Laboratory findings indicated that the carbohydrate antigen 19-9 level was within the normal limit (11.3 U/ml), but the carcinoembryonic antigen level was slightly high (7.2 ng/ml). A colonoscopy showed no tumorous lesions. Contrast-enhanced CT revealed a 63 × 55-mm well-defined, homogeneous cystic mass in the left dorsal side of the rectum and ventral side of the coccyx. Magnetic resonance imaging (MRI) showed a unilocular cystic tumor with a low signal intensity on T1-weighted images, high signal intensity on T2-weighted images, and high signal intensity on diffusion-weighted images (Fig. 1). The tumor was diagnosed as a developmental cyst based on the radiological findings, and laparoscopic resection was performed.

**Fig. 2:**
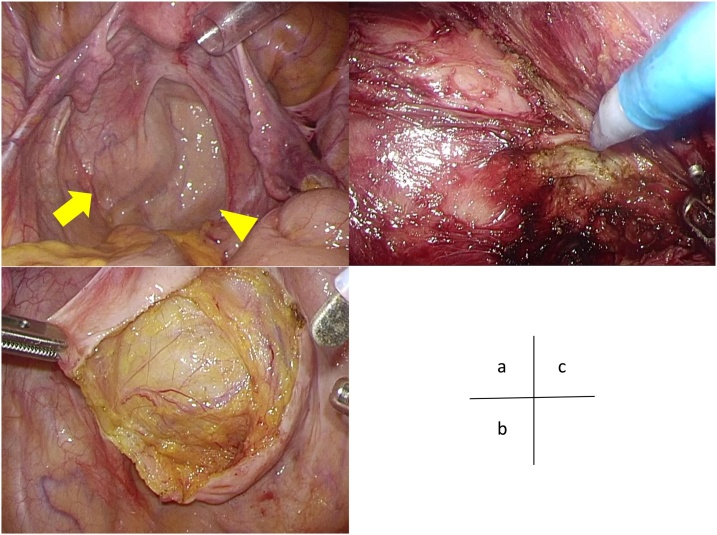
Laparoscopic findings in the pelvic cavity. (a) The arrow indicates the tumor, which is located on the left side of the rectum (arrowhead). (b) The arrow indicates the retrorectal epidermoid cyst as a retroperitoneal tumor. (c) The epidermoid cyst is a well-defined lesion, and ablation between the tumor and surroundings was easy to perorm.

The patient was placed in the lithotomy position. Access to the tumor was gained with by using a 10-mm umbilical port with the Hasson technique, followed by four 5-mm ports in both the right upper/lower and left upper/lower quadrants. The uterus was fixed to the anterior abdominal wall with sutures. The tumor was placed along the left lateral side of the rectum (Fig. 2(a)). When the peritoneum was incised, a capsulated tumor was observed, and the caudal side of the tumor continued up to the level of the levator -ani (Fig. 2(b)). Resection around the whole circumference of the tumor was performed by using the Harmonic and the electric scalpel without adhesion to the surroundings (Fig. 2(c)). There was no injury to the rectum. The operating time was 192 min, and total blood loss was 30 ml.

**Fig. 3:**
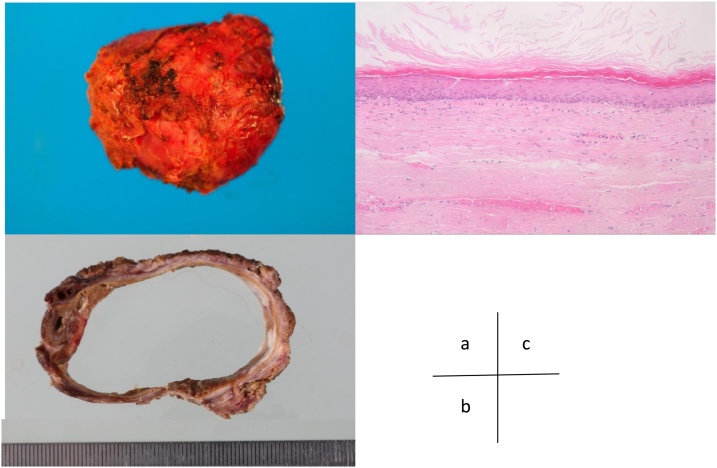
Histopathological findings of the resected tumor. (a) The specimen measures 60 × 50 mm, and it is well circumscribed and soft. (b) The cut section of the gross specimen shows a thin-walled cyst filled with yellowish-colored gelatinous materials. (c) Microscopic findings show that the wall of cyst is lined with stratified squamous epithelia with cutaneous appendages.

Macroscopically, the tumor formed a 40 × 35 × 30-mm cystic wall filled with mucoid, yellowish-colored fluid. Microscopically, the inner surface of the cyst was lined with stratified squamous epithelia (Fig. 3(a)–(c)). The histopathologic diagnosis was an epidermoid cyst, and there was no evidence of malignancy.

No postoperative event occurred, and the patient was discharged on postoperative day 5. Six months postoperatively, there was no signs of recurrence.

## 3 Discussion

The presacral cavity is the space bounded by the rectum in the anterior direction, sacrum/coccyx on the posterior side, peritoneal reflection in the cranial margin, and anterior sacrococcygeal ligament and coccygeus muscle on the caudal [4]. Presacral tumors are uncommon with an incidence of 1/40,000–63,000 patients [5,6] and 60% of presacral tumors are congenital [5].

The term “developmental cysts” was defined as presacral congenital cystic tumors produced by developmental error in the embryonal phase, and these cysts were thought to arise from caudal embryonic vestiges [7]. They occur mostly in middle-aged women [1]. Developmental cysts can be pathologically divided into epidermoid cysts, dermoid cysts, or tailgut cysts [7]. Both epidermoid cysts and dermoid cysts are lined with stratified squamous epithelia, but dermoid cysts also contains skin appendages. Tailgut cysts are lined with various epithelia, such as columnar cells, squamous cells, and transitional cells [1,7]. Malignant transformation of developmental cysts is rare, but it has been reported in the literature [8].

CT and MRI are useful in diagnosing presacral tumors. It has been reported that a low signal intensity appears on a T1-weighted image and high signal intensity appears on a T2-weighted image. Epidermoid cysts contain fatty elements, such as desquamated debris, cholesterol, keratin, and water [9]. A preoperative biopsy should not be performed because it can lead to tumor dissemination, abscess, fecal fistula, or meningitis [5].

Epidermoid tumors are located anatomically deep within the pelvis, and they are difficult to reach. Therefore, they have been traditionally accessed through sacral, abdominal, or a combined abdomino-sacral approach. The best approach is determined in consideration of the size and location of the tumors. An abdominal approach is preferred in the case of tumors developing on the cranial side. It is safe to use an abdominal approach to recognize locate the anatomical component in the pelvis easily, but this approach is more invasive because it requires a larger incision. If the tumor is small (≤10 cm), located at the caudal level (below S4), and has not invaded surrounding structures, a sacral approach is often selected [10]. This approach is less invasive than the abdominal approach, but it is provides limited surgical space. If the coccyx and sacrum are partially excised, there is a risk that pain will remarkably persist postoperatively. Furthermore, since the incision wound is near the anus, the risk of wound infection is considered to be increased [11].

In our patient, there was no difficulty in the field of view and forceps operability during laparoscopic surgery. Since an epidermoid cyst is a well-defined lesion, ablation between the tumor and muscle is easy because of the magnifying effect of the laparoscope. Furthermore, it is possible to perform laparoscopic surgery with minimal damage to the muscles, nerves, and rectum, leading to the preservation of anal function. However, a longer operating time may be required.

Laparoscopic surgery is considered to be one of the options in terms of its minimal invasiveness, reduced risk of complications, and complete tumor removal. Laparoscopic resection of an epidermoid cyst might be implemented in carefully selected cases with consideration of the tumor size and location.

## Conflicts of interest

There are no conflicts of interest.

## Funding

There are no study sponsors or sources of funding.

## Ethical approval

No ethics committee approval is required at our institution for a case report involving a single patient.

## Consent

Written informed consent was obtained from patient for publication of this case report and accompanying images.

## Author contribution

1 Koichi Mohri wrote the paper and did data collection and inter-pretation.
2 Tadahiro Kamiya, Kazuhiro Hiramatsu, Yoshihisa Shibata, Motoi Yoshihara, Taro Aoba, Akira Ito, and Takehito Kato reviewed this article and approved.

## Registration of research studies

Not applicable.

## Guarantor

Koichi Mohri.

## Provenance and peer review

Not commissioned, externally peer-reviewed.

## References

[1] Dahan H., Arrivé L., Wendum D. (2001). Retrorectal developmental cysts in adults: clinical and radiologic histopathologic, differential diagnosis, and treatment. Radiographics.

[2] Zhou J.L., Wu B., Xiao Y. (2014). A laparoscopic approach to benign retrorectal tumors. Tech. Coloproctol..

[3] Agha R.A., Borrelli M.R., Farwana R. (2018). The SCARE 2018 statement: updating consensus Surgical CAse REport (SCARE) guidelines. Int. J. Surg..

[4] Jarboui S., Jarraya H., Mihoub M.B. (2008). Retrorectal cystic hamartoma associated with malignant disease. Can. J. Surg..

[5] Jao S.W., Beart R.W., Spencer R.J. (1985). Retrorectal tumors: mayo clinic experience, 1960–1979. Dis. Colon Rectum.

[6] Lev-Chelouche D., Gutman M., Goldman G. (2003). Presacral tumors: a practical classification and treatment of a unique and heterogeneous group of diseases. Surgery.

[7] Hawkins W.J., Jackman R.J. (1953). Developmental cysts as a source of perianal abscesses, sinuses and fistulas. Am. J. Surg..

[8] Abel M.E., Nelson R., Prasad M.L. (1985). Parasacrococcygeal approach for the resection of retrorectal developmental cysts. Dis. Colon Rectum.

[9] Hannon J., Subramony C., Scott-Conner C.E. (1994). Benign retrorectal tumors in adults: the choice of operative approach. Am. Surg..

[10] Akbulut S. (2013). Unusual cause of defecation disturbance: a presacral tailgut cyst. Eur. Rev. Med. Pharmacol. Sci..

[11] Buchs N., Taylor S., Roche B. (2007). The posterior approach for low retrorectal tumors in adults. Int. J. Colorectal Dis..

